# Human Amniotic Membrane Procurement Protocol and Evaluation of a Simplified Alkaline Decellularization Method

**DOI:** 10.3390/mps9010005

**Published:** 2026-01-01

**Authors:** David A. de la Garza Kalife, Antonio Rojas Murillo, Rodolfo Franco Marquez, Diana Laura Morales Wong, Jorge Lara Arias, José Felix Vilchez Cavazos, Hector Leija Gutierrez, Mario A. Simental Mendía, Elsa Nancy Garza Treviño

**Affiliations:** 1 Department of Biochemistry and Molecular Medicine, School of Medicine, Universidad Autónoma de Nuevo Leon, Monterrey 64460, Nuevo León, Mexico; david.delagarzaka@uanl.edu.mx (D.A.d.l.G.K.); juan.rojasmrll@uanl.edu.mx (A.R.M.); 2 Department of Anatomic Pathology and Cytopathology, University Hospital “Dr. José Eleuterio González”, School of Medicine, Universidad Autónoma de Nuevo Leon, Monterrey 64460, Nuevo León, Mexico; rodolfo.francomrqz@uanl.edu.mx (R.F.M.); diana.moraleswng@uanl.edu.mx (D.L.M.W.); 3 Orthopedic Trauma Service, University Hospital “Dr. José Eleuterio González”, School of Medicine, Universidad Autónoma de Nuevo Leon, Monterrey 64460, Nuevo León, Mexico; jorge.larars@uanl.edu.mx (J.L.A.); jose.vilchezcvz@uanl.edu.mx (J.F.V.C.); 4 Center for Research in Physical and Mathematical Sciences, Universidad Autónoma de Nuevo Leon, San Nicolas de los Garza 66455, Nuevo León, Mexico

**Keywords:** amniotic membrane, decellularization, extracellular matrix preservation

## Abstract

Amniotic membrane (AM) has gained wide application in regenerative medicine due to its biocompatibility and extracellular matrix (ECM) composition. Effective decellularization is essential to minimize immunogenicity while preserving tissue architecture. This study standardized AM procurement and compared a simplified alkaline-based decellularization protocol with a conventional detergent–alkaline method, emphasizing practicality, histological integrity, and collagen preservation. Methods: Human AM was aseptically obtained from placental tissue and processed using either method. Histological analysis with hematoxylin eosin and Masson’s trichrome staining quantified nuclear content and collagen integrity. Results: The alkaline method achieved the greatest nuclear clearance but retained epithelial outlines, indicating partial persistence of cellular structures. In contrast, the detergent method achieved complete morphological decellularization but showed slightly higher residual nuclear signal. Masson’s trichrome staining revealed that the detergent-based method preserved collagen intensity most closely to native tissue (mean gray values: 128.3 ± 28.2 vs. 140.2 ± 23.4), while the alkaline group exhibited significantly reduced staining (177.8 ± 17.2; *p* < 0.001). Conclusions: the simplified alkaline method provided efficient decellularization with reduced cost, time, and cytotoxic risk, making it a practical approach for AM processing. However, partial ECM alteration suggests that detergent-based methods remain preferable when optimal structural preservation is required.

## 1. Introduction

The amnion is a thin, avascular, translucent fetal membrane that lines the amniotic cavity and covers the chorionic plate. It consists of a single epithelial layer of cuboidal to low columnar cells derived from the fetal ectoderm, supported by a basement membrane and an underlying mesodermal layer of compact stroma and fibroblasts [1]. The stromal components include a dense, compact layer rich in types I and III collagen, as well as a fibroblastic layer containing amniotic mesenchymal stromal cells that secrete types I, III, V, and VI collagen into the extracellular matrix (ECM) [2]. Although closely apposed to the chorionic fetal membrane, it is only attached via an intermediate spongy layer composed of loosely arranged collagen fibers, proteoglycans, and glycoproteins that facilitate separation from the underlying chorion [1,2]. The amnion contributes to amniotic sac integrity, amniotic fluid homeostasis, and serves as a source of bioactive components with therapeutic potential.

Amniotic membrane (AM) has been used in regenerative medicine for decades, with established applications in ophthalmology, dermatology, and other surgical fields, including artificial corneas, ocular surface reconstruction, skin wound healing, adhesion prevention, vascular grafts, connective tissue regeneration, and cartilage tissue engineering [3,4]. The reasoning behind these uses includes mechanical protection, promotion of epithelialization, anti-inflammatory, anti-fibrotic, anti-angiogenic, and antimicrobial effects [5,6].

The AM’s distinctive biological properties are mediated by its secretion of cytokines and growth factors, which modulate immune responses, inhibit fibrosis, and promote cell migration and wound healing [7,8]. Quantitative and qualitative analyses have shown that the AM contains significant levels of growth factors, with their levels influenced by preparation methods, particularly the preservation of the epithelium [9]. Preserved AM with intact epithelium contains higher levels of growth factors compared to AM with removed epithelium, indicating they originate from the epithelium [9]. These extracts stimulate the proliferation of keratinocytes and fibroblasts, supporting their role in tissue regeneration.

Growth factors and cytokines present in AM-conditioned media vary depending on the preparation method [8]. Grzywocz et al. demonstrated that preparation methods affect growth factor profiles in AM-derived cell culture media, with different enzymatic treatments yielding distinct cell fractions—one mixed (amniotic epithelial cells and amniotic mesenchymal stromal cells) and one predominantly epithelial, each secreting different sets of growth factors and their receptors [10]. Litwiniuk et al. found that the content of regenerative factors in AM varies by donor, membrane region, and delivery method, highlighting the challenges in standardizing the procurement of consistent AM-based materials [11]. The release profile of these factors is highly dependent on preservation techniques, with non-preserved and cryopreserved AM maintaining higher viability and a broader spectrum of secreted factors compared to glycerol-preserved AM, which shows minimal growth factor release [12]. One major challenge in AM procurement is the scarcity of detailed, standardized protocols in the literature, which limits reproducibility and consistent tissue quality across studies.

Recent advances in AM research have focused on a variety of modifications, such as lyophilization, decellularization, and cryopreservation, to optimize its use as a biological scaffold in regenerative medicine and tissue engineering [13]. Because the AM contains epithelial cells, mesenchymal stromal cells, and fibroblasts that may trigger immunogenicity, decellularization is used to remove cells while preserving the ECM, assessed by optimal microscopy, fluorescence staining, or DNA quantitative assay [14]. Decellularization protocols typically employ enzymatic (e.g., thermolysin, trypsin), detergent-based (e.g., sodium dodecyl sulfate, Tween 80), alkaline (e.g., sodium hydroxide), or mechanical methods, often in combination [15]. Peracetic acid-based protocols have demonstrated superior preservation of ECM components and removal of cellular debris compared to other chemical methods [16]. Enzymatic and detergent-based protocols achieve effective decellularization but may require long processing times and can variably affect ECM integrity [17].

On the contrary, a simple and rapid method using 0.5 M sodium hydroxide (NaOH) applied to the epithelial side of the AM efficiently removes adherent cells within a minute, with prior reports of excellent preservation of basement membrane components such as laminin, type IV collagen, fibronectin, and perlecan [18]. Saghizadeh et al. proposed an alkaline decellularization method to remove cells using a brief chemical treatment, followed by washing steps [18]. Gholipourmalekabadi et al. [19] also employed a protocol similar to that approach and also included a short pretreatment and additional washing and drying steps to preserve the membrane. These alkaline methods are simple and low-cost, and if shown to be as effective as traditional detergent-based techniques, they could greatly reduce the time and expense involved in producing decellularized amniotic membrane (dAM).

In previously reported alkaline decellularization protocols, the amniotic membrane is cut into small fragments prior to chemical treatment. However, this step may restrict the potential applications of the final product. Therefore, we hypothesized that membrane fragmentation is not required and that the amniotic membrane can be effectively decellularized without this additional manipulation.

Given the therapeutic potential of AM and the growing interest in its use as a decellularized biomaterial, objective of this study were establish a standardized procurement for human amniotic membrane procurement followed evaluated a simplified alkaline decellularization method can remove cellular components from the amniotic membrane as effectively as the conventional detergent–alkaline protocol, while preserving extracellular matrix integrity and offering advantages in cost, processing time, and safety for future regenerative applications.

## 2. Experimental Design

The procedures include the assembly, packaging, and sterilization of materials used during amniotic membrane procurement and cleaning. All instruments and consumables are packed in sterilizable pouches and labeled for traceability before sterilization at 155 °C. Proper labeling and handling prevent confusion with already sterilized kits and ensure safe use during surgical collection.

Amniotic Membrane Procurement Kit

The following materials are required to assemble the amniotic membrane procurement kit:

− 30 cm container;
− Plastic kidney dish;
− Curved Mayo scissors;
− Surgical gown;
− Surgical drape;
− Empty storage container;
− Gauze pads;
− Surgical towel;
− Pairs of surgical gloves (a pair half a size larger than the other).

Amniotic Membrane Cleaning Kit

The following materials are required to assemble the amniotic membrane cleaning kit:

− Gauze pads
− Toothless dissection forceps
− Mayo tray
− Plastic mesh
− Self-sealing pouch (13.5 cm × 25.5 cm).

Solutions for Amniotic Membrane Processing

The following materials are required to clean the amniotic membrane:

− 200 mL of sterile water;
− 1 L Saline solution (0.9%) with antibiotic–antimycotic cocktail (including 100 μg/mL gentamicin);
− 2 L PBS containing antibiotic–antimycotic cocktail (including 100 μg/mL gentamicin).

Solutions for Amniotic Membrane Decellularization

The following materials are required to decellularize the amniotic membrane:

− 500 mL 0.1 M NaOH;
− 500 mL 0.5 M NaOH;
− 500 mL 0.1%Tween 80;
− 500 mL 0.15% peracetic acid in ethanol (96% *v*/*v*);
− 500 mL PBS.

## 3. Procedure

### 3.1. Donor Selection and Informed Consent

Placentas were obtained with ethical approval from the University Hospital “Dr. José E. González” Ethics Committee, granted under record number BI23-00002. AM donor candidates were identified based on predefined inclusion and exclusion criteria. Clinical records of patients scheduled for cesarean section were reviewed to confirm eligibility. Donors were required to be over 18 years old, under 40 weeks of gestation, and have no significant medical complications during pregnancy or delivery. Exclusion criteria were applied following the guidelines described by Fraga et al. to ensure sample quality and consistency such as lacking placental abruption or membrane rupture at the time of the cesarean section, preeclampsia, gestational diabetes, or maternal infections [20]. Once eligible donors were selected, the AM donation process was explained, and informed consent was obtained in accordance with the Declaration of Helsinki and institutional ethical guidelines.

### 3.2. Placenta Procurement

1. Collect the placenta (*n* = 12) immediately after cesarean delivery under aseptic conditions.
2. Place the placenta into a sterile bowl together with a disposable umbilical clamp.
3. Position the placenta with the fetal surface (continuous with the umbilical cord) facing upward.
4. Clamp the umbilical cord 1–2 cm above the placental surface using the disposable clamp.
5. Compress the umbilical cord with sterile gauze to drain remaining blood and cut above the clamp using Mayo scissors.

#### 3.2.1. Rinsing and Surface Cleaning

1. Rinse the placenta multiple times with sterile saline or an antibiotic–antimycotic solution (penicillin, streptomycin, neomycin, and amphotericin B) to eliminate bacteria and fungi [21].
2. Clean the fetal surface gently with sterile gauze to remove blood and residual tissue debris.
3. Place the placenta fetal side up in a sterile container lined with a laparotomy sponge to absorb maternal blood and maintain a clean working area.

#### 3.2.2. Amniotic Membrane Separation

**Figure 1:**
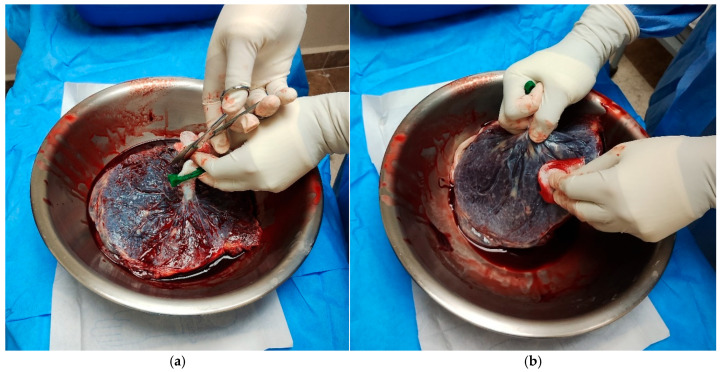
Macroscopic preparation of the placenta for AM procurement. (**a**) The umbilical cord is cut over a sterile surgical bowl to facilitate handling and exposure of the fetal membranes. (**b**) The superficial surface of the attached AM is gently cleaned using gauze to remove blood and debris, ensuring better visualization of the tissue layers for separation.

**Figure 2:**
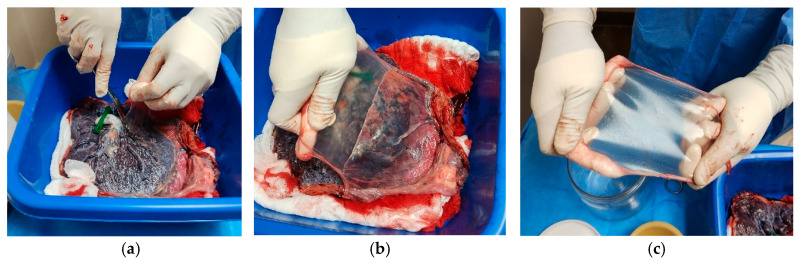
Stepwise isolation of the AM from the placenta. (**a**) Using scissors, an initial incision is made around the umbilical cord to facilitate membrane separation. (**b**) The fetal membranes are gently separated in a lateral fashion by blunt dissection. (**c**) The isolated AM is held and stretched, revealing its translucent appearance and structural integrity.

**Figure 3:**
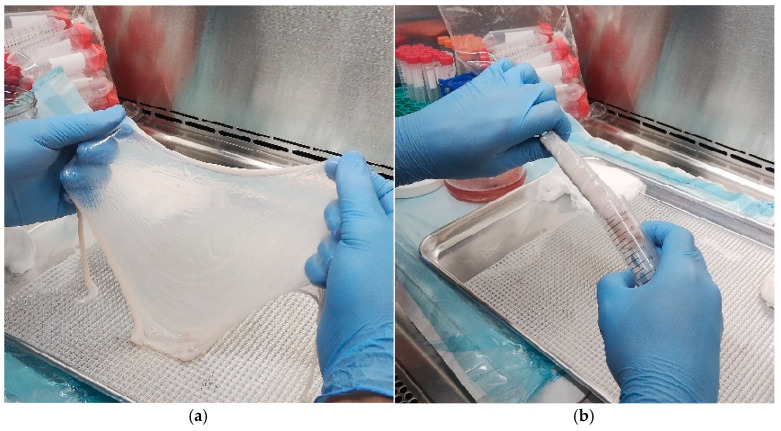
Processing and storage of the AM inside the laminar flow hood. (**a**) The AM is held up and gently stretched during the cleaning process to ensure the removal of blood and debris. (**b**) The cleaned membrane is rolled with the epithelial surface facing outward and placed into a sterile 50 mL tube for storage.

1. Identify the amniotic membrane as the translucent layer continuous with the umbilical cord epithelium.
2. Locate a natural separation plane between the amniotic membrane (AM) and chorion if present.
3. Make a small incision with sterile scissors when no natural separation plane exists to initiate membrane separation, avoiding excessive tension to prevent tearing [22,23].
4. Separate the AM from the chorion by gently lifting and dissecting the membrane.
  Note: Process placentas with central cord insertion by separating the membrane centripetally, moving from the periphery toward the cord.
  Process placentas with eccentric cord insertion by first cutting around the cord and then lifting and separating the membrane laterally.

5. Perform careful dissection in areas with generalized rupture to avoid tissue damage (Figure 1).
6. Cut the AM around the umbilical cord using Mayo scissors to free the membrane from its attachment.
  Note: The membranes appeared uniformly thin, elastic, and transparent, with minimal residual blood following thorough rinsing with sterile saline (Figure 2).

7. Roll the amniotic membrane (AM) carefully with the epithelial surface facing outward around sterile gauze.
8. Place the rolled membrane into a sterile 50 mL tube in sterile saline or PBS (Figure 3).

#### 3.2.3. Rising and Surface Cleaning

1. Introduce the container with the AM and an isotonic rinsing solution to a laminar flow hood.
2. Transfer the membrane with sterile forceps onto a sterile mesh-lined tray and rinse thoroughly on both sides using the rinsing solution [24].
3. Inspect the AM macroscopically to exclude any visible anomalies and confirm tissue integrity.
4. Roll the AM around gauze with the epithelial surface facing outward and store in a 50 mL sterile tube.
5. Label the tube with the procurement date, the donor’s full name, and their medical record number.
6. Use fresh AM soaked in antimicrobial solution only for short-term applications, noting its limited sterility and short shelf life.
7. Freeze the AM at −28 °C (up to 8 months) or −80 °C for long-term preservation (up to 2 years), ensuring access to appropriate freezing equipment and pretreatment steps prior to use [25].
8. Assign the membranes to two different decellularization protocols (*n* = 5 total) according to the comparative study design.

#### 3.2.4. Decellularization

##### Alkaline Decellularization Protocol (Modified from Saghizadeh et al. [18])

1. Thaw the frozen amniotic membranes stored at −80 °C by immersing them in PBS for 10–30 min at room temperature.
2. Place each membrane with the epithelial surface facing upward.
3. Soak a cotton-tipped applicator in 0.5 M NaOH.
4. Gently rub the epithelial surface with the NaOH-soaked applicator to initiate epithelial removal.
5. Immerse the membrane in 0.5 M NaOH for 20–30 s.
6. Transfer the membrane immediately to PBS to stop the alkaline reaction.
7. Wash the membrane in PBS two to three times, each wash lasting 10 to 15 min.
8. Maintain gentle agitation during all washes to ensure uniform treatment and prevent structural damage.

##### Detergent Alkaline Decellularization Protocol (Modified from Villamil-Ballesteros et al. [26])

1. Thaw the frozen amniotic membranes at room temperature for 2 h.
2. Immerse each membrane in 0.1% Tween 80 for 4 h with continuous mechanical stirring.
3. Transfer the membrane to 0.1 M NaOH and incubate for 1 h.
4. Prepare an acid solution consisting of 0.15% peracetic acid in ethanol (96% *v*/*v*).
5. Soak the membrane in the peracetic acid/ethanol solution for 12 h under mechanical stirring.
6. Wash the membrane in 70% ethanol for 1 h.
7. Buffer the membrane in PBS for 2 h with agitation.
8. Immerse the membrane again in 0.1 M NaOH for 1 h.
9. Transfer the membrane back into 0.15% peracetic acid for 1 h.
10. Wash the membrane in PBS four times to remove residual chemicals.
11. Remove residual ethanol completely by performing three additional PBS washes, 2 h each.
12. Store the processed membrane at −80 °C.
  Note: Maintain gentle agitation throughout all steps to ensure homogeneous chemical exposure and to minimize ultrastructural damage. Can apply optional antimicrobial or irradiation steps as needed to enhance disinfection, consistent with detergent-based decellularization practices

A total of four amniotic membranes were processed individually by using the decellularization protocols, each immersed in approximately 500 mL of solution to ensure complete coverage. An additional membrane was immediately fixed in 4% neutral-buffered formalin and used as the native AM control. All steps were performed in sterile 3 L glass beakers, as required to allow the membrane to lie flat without folding. Sterile PBS and freshly prepared NaOH solutions were used. Thawing, alkaline or detergent treatment, and washing were carried out inside a Class II biological safety cabinet to maintain sterility and prevent cross-contamination.

### 3.3. Histology

1. Fix tissue samples (native AM and decellularized AMs) in 4% neutral-buffered formalin for 24 h at room temperature.
2. Dehydrate samples through a graded ethanol series.
3. Clear samples in xylene.
4. Embed samples in paraffin.
5. Cut paraffin sections at 4 µm thickness using a microtome and mount sections on glass slides.
  Note: For each sample in all groups (two biological replicates, each stained in duplicate), three tissue sections were analyzed per slide, and eight non-overlapping high-power fields (HPFs) were randomly selected for evaluation.

Hematoxylin and eosin (H&E) staining was used to assess the presence and condition of the amniotic epithelial cells, stroma, and tissue structure to confirm the absence of nuclei and cellular remnants as cited by Gómez et al. [24]. Masson’s trichrome staining was used to evaluate the preservation and distribution of collagen and other ECM components. Stained sections were examined under light microscopy. Representative images were captured for qualitative comparison between native AM and decellularized samples (dAMs), as well as between the two decellularization protocols.

### 3.4. Statistical Analysis

Histological quantification from H&E and Masson’s trichrome staining was performed to compare cellularity and ECM preservation across three groups: native AM, detergent dAM, and alkaline dAM. For each sample in all groups (two biological replicates by duplicate in each staining), eight non-overlapping high-power fields (HPFs) were randomly selected for analysis. Nuclei count per HPF was determined from H&E-stained sections using ImageJ (version 1.54p, Fiji distribution) to assess residual cellular content following a standardized processing pipeline: images were converted to 8-bit (Image → Type → 8-bit), thresholded (Image → Adjust → Threshold; 0–110, De-fault, Red, “Don’t reset range” checked), scale deleted and selection cleared, followed by median filtering (Process → Binary → Median; radius 3). Particle analysis was then performed (Analyze → Analyze Particles; size 30–Infinity, circularity 0.30–1.00, “Summarize” and “Overlay” checked) to obtain nuclei counts [27]. To quantify collagen staining intensity, Masson’s trichrome-stained images were processed using ImageJ’s Color Deconvolution plugin (Image → Color → Color Deconvolution), retaining only the blue channel specific to collagen. Measurements were configured (Analyze → Set Measurements; Area, Standard Deviation, and Mean Gray Value checked), and mean gray values were obtained from selected regions (Analyze → Measure). These quantitative metrics enabled direct comparison of decellularization efficacy and ECM integrity between treatment groups.

Data were expressed as mean ± standard deviation. Normality was assessed using the Shapiro–Wilk test. For normally distributed data, one-way ANOVA followed by Tukey’s post hoc test was used to compare differences among groups. For non-normally distributed data, the Kruskal–Wallis test followed by Dunn’s post hoc test was applied. A *p*-value < 0.05 was considered statistically significant. All statistical analyses were conducted using GraphPad Prism (version 5.03).

## 4. Results

*Histological Evaluation*

H&E staining revealed a progressive reduction in cellular content across native AMs, detergent dAMs, and alkaline dAMs. Native membranes exhibited a continuous epithelial layer with abundant, densely stained nuclei, as well as scattered nuclei in the stroma, confirming the presence of intact amniotic epithelial cells and mesenchymal stromal cells. Detergent-treated membranes displayed near-complete removal of cellular structures, with no morphologically visible nuclei or cell architecture under light microscopy. In contrast, alkaline-treated membranes showed visibly reduced nuclear density, but epithelial cell outlines and residual nuclear debris were still apparent, indicating partial decellularization.

**Figure 4:**
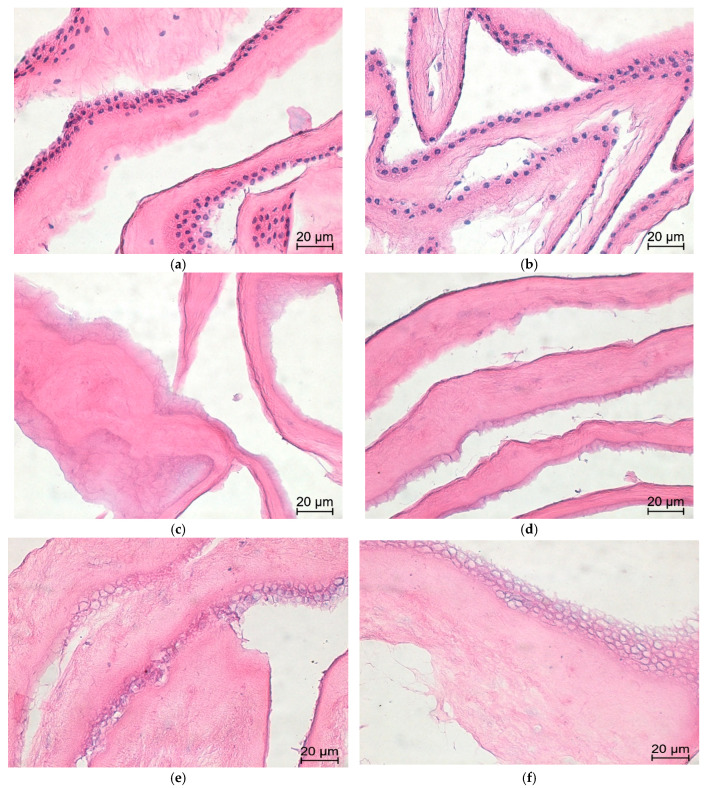
Representative H&E-stained images of human AM at 40× magnification. (**a**,**b**) Native membranes show abundant hematoxylin-positive nuclei and intact epithelial and stromal layers. (**c**,**d**) Detergent dAMs exhibit near-complete removal of nuclei with preserved overall morphology. (**e**,**f**) Alkaline dAMs show marked nuclear clearance but retain faint outlines of epithelial structures, suggesting residual cellular architecture.

Quantitative image analysis of hematoxylin-positive material yielded average nuclei counts of 84 ± 34 per HPF in native membranes, 10 ± 7 nuclei/HPF in the detergent group, and 3 ± 6 nuclei/HPF in the alkaline group. Statistical analysis using the Kruskal–Wallis test followed by Dunn’s post hoc test revealed significant differences between the native AM and both dAM groups (*p* < 0.01), confirming effective reduction in nuclear material (Figure 4). However, no significant difference was observed between the detergent-based and alkaline decellularization methods. These results were validated by two additional independent observers, who performed a manual count of the nuclei and obtained similar values as shown in Table 1.

**Figure 5:**
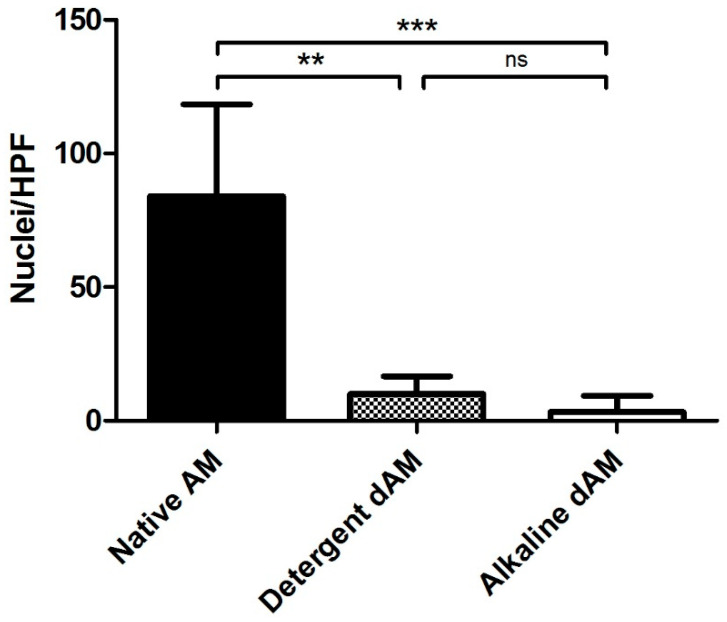
Quantification of nuclei per HPF in native, detergent-decellularized, and alkaline-decellularized AMs. Bars represent the mean ± standard deviation (*n* = 2 biological replicates per group, 8 HPFs per sample. Both decellularization methods significantly reduced nuclear content compared to native tissue (** *p* < 0.05; *** *p* < 0.001, Kruskal–Wallis with Dunn’s post hoc test), with no significant difference between detergent and alkaline treatments.

Quantification was performed using eight HPFs per sample across *n* = 2 biological replicates per group. Representative histological images are shown in Figure 5. Although the alkaline group showed the lowest hematoxylin-positive signal, morphological evaluation revealed persistent epithelial outlines, suggesting incomplete structural clearance. Conversely, detergent dAMs appeared cleaner under microscopy, with no visible nuclei or outlines, despite slightly higher hematoxylin-positive values, likely due to sub-threshold residual nuclear fragments.

**Figure 6:**
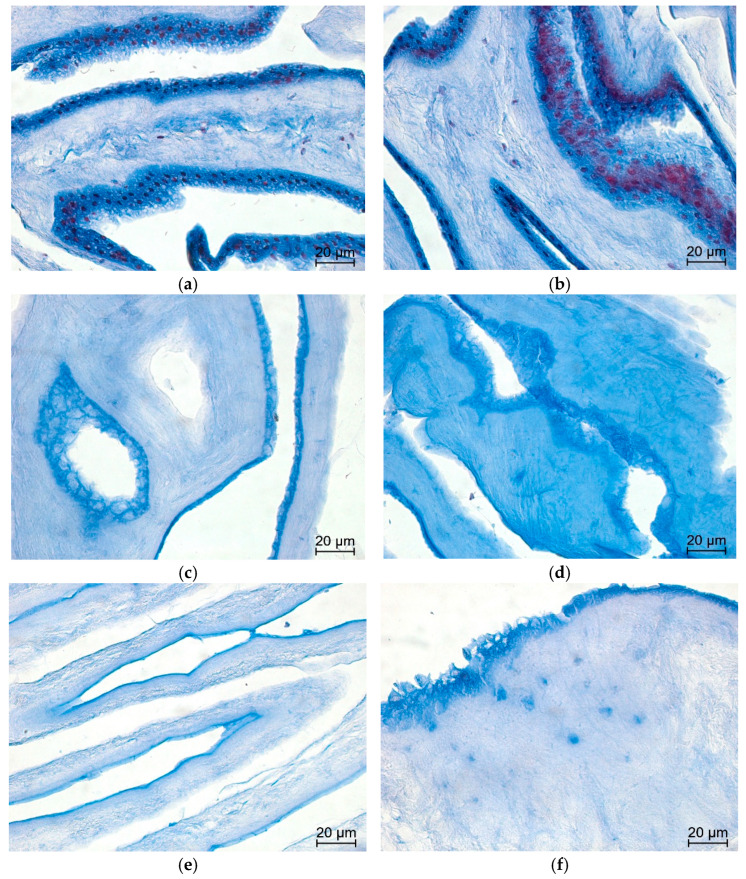
Representative Masson’s trichrome-stained images of human AM at 40× magnification. (**a**,**b**) Native membranes display dense, continuous collagen fibers with intense blue staining, indicating an intact ECM structure. (**c**,**d**) Detergent dAMs retain uniform collagen distribution and strong staining intensity, reflecting preserved matrix integrity. (**e**,**f**) Alkaline dAMs exhibit slightly reduced collagen staining and mild disruption in matrix organization, suggesting partial alteration of ECM structure.

Masson’s trichrome staining was used to evaluate collagen preservation and ECM integrity across all groups. Quantitative analysis of mean gray values showed that the detergent-based method produced the darkest collagen staining (128.3 ± 28.2), followed by native tissue (140.2 ± 23.4) and the alkaline group (177.8 ± 17.2), suggesting comparable collagen preservation despite varying degrees of decellularization (Figure 6).

**Figure 7:**
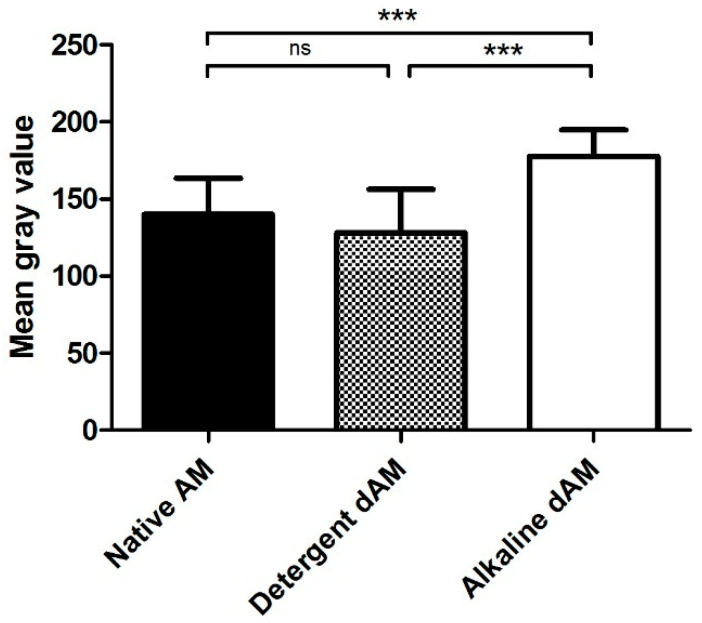
Quantification of collagen staining intensity by mean gray value from Masson’s trichrome-stained sections of native, detergent dAM, and alkaline dAM. Bars represent the mean ± standard deviation (*n* = 2 biological replicates per group, 8 HPFs per sample). One-way ANOVA with Tukey’s post hoc test showed a significant decrease in collagen intensity in the alkaline group compared to both native and detergent groups (*** *p* < 0.001), while no significant difference (ns) was observed between the native and detergent-treated samples.

One-way ANOVA with Tukey’s post hoc test revealed statistically significant differences between the alkaline dAM group and the other groups (*p* < 0.001), indicating collagen loss associated with this method. No significant difference was observed between the native AM and the detergent dAM group in terms of collagen staining intensity. Microscopically, the native membrane exhibited densely packed, continuous collagen fibers; the detergent group preserved similar staining intensity and structure, while the alkaline group showed visibly reduced collagen intensity, suggesting partial ECM degradation (Figure 7).

## 5. Discussion

This study aimed to compare the effectiveness of two decellularization protocols—alkaline and detergent-based—by assessing AM cellularity and ECM preservation using histological evaluation. Both methods significantly reduced cellular content, as confirmed by quantitative H&E staining. However, histological and image-based analyses revealed mixed outcomes, with distinct advantages and limitations for each protocol.

Procurement observations highlighted membrane rupture and contamination risk, consistent with previous reports. As suggested by Khosravimelal et al., careful separation of the amnion from the chorion and strict aseptic technique are critical to preserving tissue integrity and sterility throughout processing [17]. Our protocol addressed these challenges through immediate post-delivery collection, aseptic handling under laminar flow conditions, and controlled dissection techniques, which collectively minimized mechanical damage and microbial exposure. The consistent identification of the amnion based on translucency and elasticity, along with systematic rinsing and standardized rolling for storage, ensured reproducible tissue quality across samples. This in-depth description of our procurement method contributes meaningfully to the literature by offering a replicable, stepwise guide that other researchers can follow or adapt.

Fresh native membranes served as essential controls, confirming intact epithelial and stromal morphology with dense cellularity. While the alkaline method demonstrated greater nuclear clearance, the detergent method showed more complete morphological decellularization. These divergent results suggest that the optimal protocol may depend on the specific application: the alkaline method may be better suited for uses prioritizing reduced toxicity and immunogenicity, while the detergent method may be preferred for full morphological decellularization.

The alkaline method demonstrated the greatest reduction in hematoxylin-positive nuclear material. Yet, persistent epithelial outlines were visible under light microscopy, indicating incomplete structural clearance despite substantial nuclear loss. Additionally, this group showed the lowest collagen staining intensity in Masson’s trichrome analysis, suggesting potential disruption or loosening of the stromal ECM. In contrast, the detergent-based method achieved near-complete morphological decellularization, with no visible nuclei or cellular structures, and preserved a more uniform collagen architecture microscopically. Nonetheless, it retained slightly more hematoxylin-positive signal, likely reflecting residual nuclear fragments below the threshold of visual detection.

Although the alkaline approaches described by Saghizadeh et al. and Gholipour-Malekabadi et al. [18,19] offer simple, low-cost, and less toxic protocols with the potential to preserve ECM structure, several refinements are still required to achieve complete structural decellularization. Notable methodological differences also exist between the protocol proposed by Saghizadeh et al. and the method evaluated in our study. Their procedure combines a distinct preservation strategy for the amniotic membrane (AM), either cut into small pieces and cryopreserved in PBS with 10% dimethyl sulfoxide or used fresh with the application of two NaOH concentrations for cell removal, yielding near-complete cellular elimination. In contrast, the incomplete cell removal observed in our protocol may be attributed to the larger membrane surface area exposed during treatment.

The protocol described by Gholipour-Malekabadi et al. employs three chemical agents: 0.2% EDTA, 0.5 M NaOH, and 5% NH_4_Cl, resulting in a more aggressive, multi-step procedure that requires vigorous scraping and lyophilization, which increases the risk of ECM damage. In comparison, the alkaline protocol is simpler, faster, and more likely to preserve the structural integrity of the membrane.

The other evaluated protocol (detergent–alkaline protocol) was proposed by Villamil-Ballesteros [26] and had previously been used by our research group [28]. In our previous results, a thermal-shock step followed by lyophilization and pulverization was included prior to processing, removing more than 90% of the cellular component while preserving collagen, as confirmed by immunohistochemistry. Although the thermal shock step was not a step in the protocol evaluated, our results indicate that both methods used here remain effective in achieving morphological clearance, although low-level nuclear material may persist. Without requiring thermal shock or membrane fragmentation, both protocols removed the cellular component by ≥90% in the alkaline treatment and approximately 93% in the combined method based on H&E analysis. These findings suggest that the pre-treatment step contributes only about 10–15% to overall cellular removal.

Limitations of this study include the relatively small sample size and reliance on histological and quantitative image analyses without further molecular or biomechanical assessments. Future research should incorporate proteomic characterization to evaluate ECM protein composition and growth factor retention, immunohistochemical staining to better define matrix component preservation, and mechanical testing to assess functional scaffold properties post-decellularization. Prior studies have employed diverse mechanical assessment methods, including uniaxial tensile testing with an Autograph AGS-X system [15], micrometer gauge measurements [24], and gram-range load sensors [29]. Such a comprehensive analysis will clarify the biomechanical and clinical potential of these decellularization methods.

In summary, both alkaline and detergent-based protocols significantly reduced cellular content in human AMs, but neither demonstrated unequivocal superiority across all parameters. The choice of decellularization method should be guided by the intended downstream application and the relative importance of ECM preservation versus complete morphological clearance.

## 6. Conclusions

This study evaluated and compared two decellularization protocols for human AM: a conventional detergent-alkaline method and a simplified alkaline-only approach. Both effectively removed cellular components, as demonstrated by significant reductions in hematoxylin-positive nuclei; however, histological analysis revealed distinct trade-offs. The alkaline method showed the lowest nuclear content but retained epithelial cell outlines, suggesting incomplete morphological clearance. In contrast, the detergent method achieved near-complete morphological decellularization but retained more hematoxylin-positive signal, likely representing nuclear remnants.

From a practical perspective, the alkaline method offered notable advantages: it used fewer reagents, shortened processing time, and avoided the cytotoxic risks linked to detergents. These benefits, combined with lower costs and technical simplicity, make it particularly valuable in low-resource or high-production settings; however, further validation with optimized and reproducible quantification methods is necessary before drawing definitive conclusions about its comparative performance.

In conclusion, both decellularization methods effectively reduced cellular content while preserving ECM components to varying degrees, but the relative efficacy of each method remains uncertain given the methodological constraints identified. Although neither protocol was definitively superior across all parameters, the alkaline method stands out as a cost-effective, accessible alternative that balances decellularization efficiency with matrix preservation, supporting its use in regenerative medicine, biobanking, and tissue engineering.

## Figures and Tables

**Table 1:** Efficiency of Amniotic Membrane Decellularization.

	Experimental Group				
	Native AM	Detergent Decellularization		Alkaline Lysis Decellularization	
	Nuclei Count	Nuclei Count	% Decellularized	Nuclei Count	% Decellularized
Observer 1	88.14	0.875	99.01 ± 1.53	1.99	98.01 ± 2.55
Observer 2	84.625	9.625	88.62 ± 7.65	1.375	98.37 ± 1.77
Observer 3	130.75	0.375	99.69 ± 0.63	1.625	98.68 ± 1.22

## Data Availability

No new publicity available datasets were generated in this study. The data supporting the findings of this study are included within in the manuscript.

## Abbreviations

The following abbreviations are used in this manuscript:

AM	Amniotic membrane
dAM	Decellularized amniotic membrane
ECM	Extracellular matrix
H&E	Hematoxylin and eosin
HBSS	Hanks’ Balanced Salt Solution
HPF	High-power field
PBS	Phosphate-buffered saline
